# Hydrogel-Gated Organic Electrochemical Transistors for Wearable and Portable Direct Detection of Aerosol-Borne Viral Antigens

**DOI:** 10.3390/gels12100916

**Published:** 2026-10-12

**Authors:** Haiyang Yu, Yuhan Lu, Bihan Guo, Jiameng Wang, Ya Zhang, Bingfang Wang, Qiuyu Yang, Lan Shen, Mengtian Shan, Feilong Wang, Daizong Ji, Yin Fang

**Affiliations:** 1 The Institute for Biomedical Engineering & Nano Science, School of Medicine, Tongji University, Shanghai 200331, China; 2 Department of Pulmonary and Critical Care Medicine, Shanghai East Hospital, School of Medicine, Tongji University, Shanghai 200120, China

**Keywords:** organic electrochemical transistor, hydrogel electrolyte, wearable and portable detection device, aerosol detection

## Abstract

Direct detection of viral antigens in respiratory aerosols is challenging because affinity-based organic electrochemical transistors (OECTs) typically rely on liquid samples. Here, a non-flowing agarose hydrogel replaced the liquid electrolyte to capture aerosols, conduct ions, and support target transport. Before OECT readout, a separate 1-min ring-electrode field-assistance step was applied to facilitate target delivery to the aptamer-functionalized gate. Under the optimized conditions, the device achieved a solution-phase concentration-based limit of detection (LoD) of 1.27 fM and a sample-to-result time of 17 min. The approach was implemented in face-mask and handheld formats with a portable wireless readout system. In nebulization-based tests using SARS-CoV-2 spike protein, the LoDs expressed in terms of the concentrations of the nebulizer source solutions were 6.89 pM and 1.19 pM for the face-mask and handheld configurations, respectively. These results demonstrate that hydrogel-mediated aerosol capture and ionic coupling, ring-electrode field assistance, and OECT transduction can be integrated to enable direct electrical detection of aerosol-borne viral antigens, supporting further development of wearable and portable respiratory screening systems.

## 1. Introduction

Respiratory viral infections remain a major public health concern [1,2]. Virus-containing aerosols contribute to airborne transmission and environmental exposure [3], and recent surveillance studies have detected respiratory viruses at low levels across different environments [4]. These particles are commonly released during breathing, speaking, and coughing [5], but may also be generated during vomiting or transported through building drainage systems [6,7]. Direct detection of viral biomarkers in air could therefore help clarify transmission routes and support rapid, non-invasive monitoring of respiratory infections.

However, direct detection of aerosol-borne viral antigens remains technically challenging [8]. Many affinity-based electronic biosensors, including organic electrochemical transistors (OECTs) and electrolyte-gated field-effect transistors, require a continuous liquid phase for molecular recognition and ionic conduction. They are therefore commonly designed and evaluated using sweat, tears, buffers, serum, or other liquid samples [9,10,11,12,13,14]. Aerosols, in contrast, are dispersed in air, delivered intermittently, and often contain low amounts of target, making them difficult to interface directly with liquid-based sensors. Current approaches either collect airborne targets by condensation, wet cyclones, filtration, or electrostatic deposition before transferring them into liquid [15,16,17,18,19], or create a confined air–liquid interface or rehydrate lyophilized sensing components at the sensing site [20,21]. The first approach adds sample-handling steps and may cause target dilution or loss, whereas the second requires reservoirs, sealed chambers, or fluid-control structures to prevent evaporation and leakage. These components increase system complexity and can be difficult to integrate into flexible devices operating under motion. Mechanical conformability, system integration, and sustainability are also important considerations for wearable healthcare electronics [20,21,22,23]. Direct aerosol sensing therefore requires a stable, air-facing interface that retains an aqueous electrolyte while supporting aerosol capture, molecular recognition, and ionic–electronic signal transduction.

Hydrogels provide a viable route to resolve this aerosol-sample–liquid-sensor interfacial mismatch. Previous studies have shown that an exposed hydrogel can directly contact respiratory virus aerosols and convert antigen-induced hydrogel responses into wireless electrical signals [24]. Water-rich hydrogel networks can retain an aqueous electrolyte while supporting ion transport and electrochemical operation [25,26]. For direct aerosol sensing, the hydrogel provides an exposed surface for aerosol deposition and a hydrated pathway for captured targets to reach the biorecognition interface. It therefore bridges airborne samples with liquid-phase bioelectronic sensing without the need for a free-flowing electrolyte. However, the hydrogel network can also hinder target transport. Protein diffusion in agarose depends on molecular size, gel concentration, steric hindrance, and interactions with the polymer network [27,28]. At low target concentrations, transport to the sensing interface may become a major factor determining the practical detection limit [29]. Previous studies have shown that applied electric fields can enhance the transport of proteins, nucleic acids, and peptides through hydrated, ionically conductive media, thereby improving molecular delivery to functional biointerfaces [30,31,32]. On this basis, a short field-assistance step was introduced to modulate transport within the hydrogel and enhance the subsequent sensing response.

In this study, we developed a hydrogel-gated OECT for direct detection of aerosol-borne viral antigens. The agarose hydrogel replaces the conventional free-flowing electrolyte and serves as an aerosol-capture matrix, ion-conducting electrolyte, and pathway for antigen transport. Viral antigens are recognized by an aptamer-functionalized Au gate, while a surrounding ring electrode provides a separate 1-min electric-field-assistance step before OECT readout. These elements perform interdependent functions: the hydrogel establishes a hydrated aerosol–sensor interface, the ring electrode enables separate electric-field-assistance, and the OECT converts antigen recognition into a drain-current response. Under optimized direct-application conditions, the platform achieved a solution-phase concentration-based limit of detection (LoD) of 1.27 fM. The sensor was further integrated into face-mask and handheld devices with a portable wireless readout system. In nebulization-based tests using SARS-CoV-2 spike protein, both configurations produced concentration-dependent responses without prior condensation, elution, or off-device liquid-phase processing. The corresponding calibration results were evaluated on the basis of the protein concentrations in the source solutions loaded into the nebulizer. This integrated approach provides a feasible route for direct detection of aerosol-borne viral antigens.

## 2. Results and Discussion

### 2.1. Device Design, Gate Functionalization, and Solution-Phase Sensing Validation of the Portable Hydrogel-Gated OECT Platform

**Figure 1:**
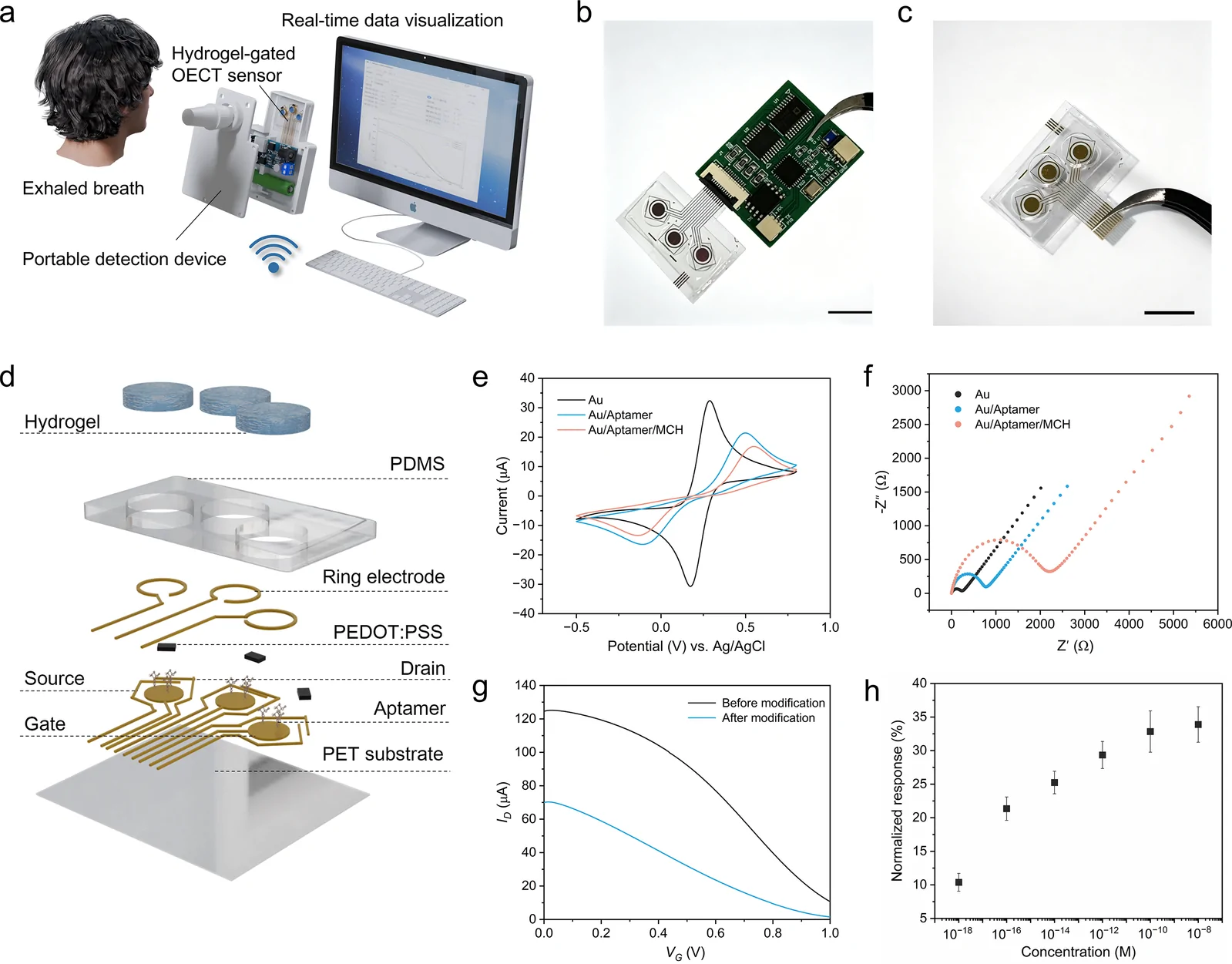
Design and validation of the portable hydrogel-gated organic electrochemical transistor (OECT) platform for aerosol-borne viral antigen detection. (**a**) Schematic of the portable sensing platform integrating aerosol sampling, electrical transduction, and real-time wireless data visualization. Photographs of the compact electronic readout module (**b**) and the OECT sensor (**c**). Scale bar, 1 cm. (**d**) Exploded schematic of the hydrogel-gated OECT sensor, comprising a Polyethylene terephthalate (PET) substrate, source, drain, gate, and ring electrode, a patterned PEDOT:PSS channel, a Polydimethylsiloxane (PDMS) well, and the hydrogel electrolyte. Cyclic voltammograms (**e**) and Nyquist plots (**f**) of the bare Au electrode and the electrode after sequential aptamer immobilization and 6-Mercapto-1-hexanol (MCH) passivation. (**g**) Transfer characteristics of the OECT before and after gate functionalization. (**h**) Normalized response of the aptamer-functionalized OECT to SARS-CoV-2 spike protein in solution (*n* = 6; mean ± SE).

Figure 1a–c illustrate a handheld sensing platform integrated with a portable electronic readout system and a hydrogel-gated OECT sensor for aerosol-borne viral antigen detection. The readout system enables real-time signal acquisition and wireless data transmission to an external computer for visualization. The hydrogel-gated OECT sensor is composed of Cr/Au electrodes, a PDMS well, and a PEDOT:PSS channel (Figure 1d). The Cr/Au electrodes, including the source, drain, gate, and ring electrode, were defined on a flexible Polyethylene terephthalate (PET) substrate using standard microfabrication processes. The PEDOT:PSS channel was subsequently patterned by a solution-based process on a lithographically defined template, yielding a channel with a width/length (W/L) of 1000 μm/70 μm (Figure S1). The gate electrode was functionalized by immobilizing aptamer probes on its surface. The PDMS well was positioned over the electrode region to confine the hydrogel electrolyte. The ring electrode was incorporated to apply a separate 1-min electric-field-assistance step before OECT readout. The hydrogel-gated OECT sensor was integrated with a custom-designed printed circuit board (PCB) for signal acquisition, wireless communication, and precise voltage control. The detachable electrode module enables convenient replacement. An external mouthpiece was used to direct the aerosol stream to the sensing interface, and a one-way valve was incorporated into the mouthpiece to allow airflow only from the user toward the sensor, thereby preventing reverse airflow from the device.

Electrochemical characterization was performed after each functionalization step to verify successful gate modification. As shown in Figure 1e, the bare Au electrode exhibited well-defined redox peaks in cyclic voltammetry (CV), indicating efficient electron transfer. Upon aptamer immobilization, the redox peak currents decreased, and further attenuation was observed after passivation with 6-mercapto-1-hexanol (MCH). This progressive suppression is consistent with the formation of an insulating self-assembled layer on the Au surface, which hinders electron transfer. Figure 1f shows the corresponding Nyquist plots of each functionalization step. The data were fitted using an equivalent circuit model (Figure S2). The bare Au electrode displayed a low charge-transfer resistance (*R_ct_*), whereas aptamer immobilization increased *R_ct_* owing to the negatively charged and insulating nature of the nucleic acid layer. A further increase in *R_ct_* after MCH treatment is attributed to the formation of a more compact blocking layer. These results are consistent with the CV data and confirm the successful formation of the functionalized interface.

The electrical response of the OECT further supports the functionalization of the gate electrode. Figure 1g shows the transfer characteristics of the device before and after gate modification, together with the corresponding normalized response toward SARS-CoV-2 spike protein (Figure 1h and Figure S3). The measurements in Figure 1h were performed using a conventional liquid electrolyte to examine the effects of ionic conditions and temperature on aptamer–target recognition. The observed signal modulation is attributed to charge accumulation at the aptamer-functionalized gate interface and interfacial potential changes induced by specific target binding. In addition, scanning electron microscopy (SEM) images reveal distinct morphological changes on the gate surface after aptamer/MCH functionalization (Figure S4), providing further evidence for successful surface modification.

The sensing performance of the aptamer-functionalized device depended strongly on Mg^2+^ and temperature. In the absence of Mg^2+^, the transfer characteristics exhibited a non-ideal shift upon target addition rather than the expected sensing response (Figure S5), indicating that Mg^2+^ is essential for maintaining the binding-competent conformation of the aptamer. In addition, the sensor response was markedly reduced at 4 °C (Figure S6), consistent with reduced binding kinetics under low-temperature conditions. Therefore, all subsequent measurements were performed in the presence of 2 mM MgCl_2_ at room temperature. Control experiments further showed negligible signal drift upon repeated addition of blank buffer and minimal response from the non-functionalized sensor toward spike protein (Figure S7), confirming a stable baseline and specific target recognition.

### 2.2. Hydrogel Concentration-Dependent Sensing Performance

**Figure 2:**
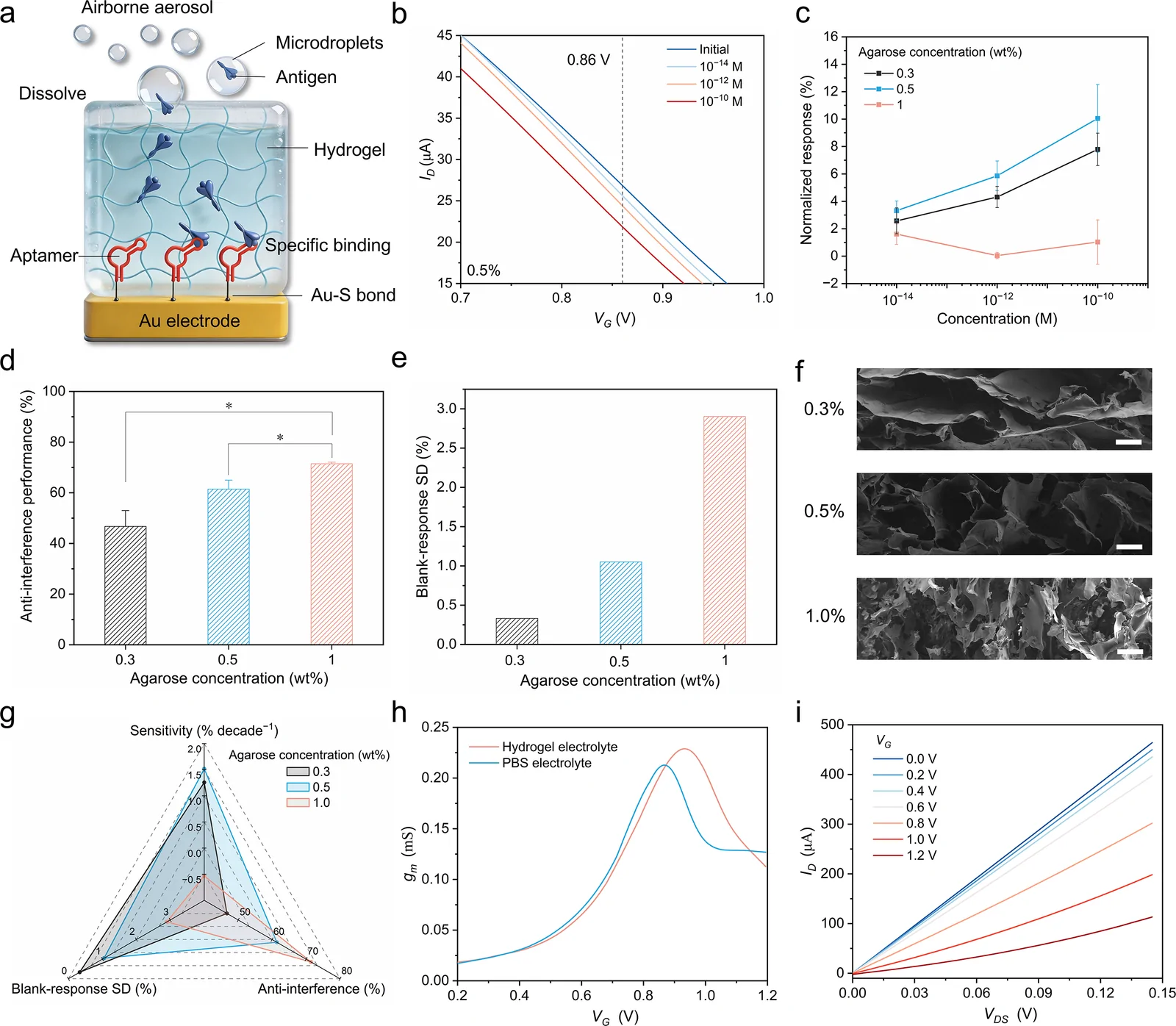
Effect of agarose concentration on the sensing performance of the hydrogel-gated OECT. (**a**) Schematic of aerosol capture and sensing at the hydrogel interface. Aerosol droplets merge with the hydrated gel matrix and release antigenic targets, which diffuse toward the aptamer-functionalized gate for specific recognition. (**b**) Representative transfer curve of the OECT incorporating 0.5 wt% agarose exposed to different concentrations of SARS-CoV-2 spike protein. (**c**) Concentration-dependent normalized responses of OECTs incorporating 0.3, 0.5, and 1.0 wt% agarose (*n* ≥ 3; mean ± SE). (**d**) Anti-interference performance of devices incorporating different agarose concentrations, with a higher value indicating a smaller artificial-saliva-induced signal perturbation (*n* = 3; mean ± SE). Brackets indicate comparisons with the 1.0 wt% group (* *p* < 0.05, two-sided unpaired *t*-test). (**e**) Standard deviation of the blank response obtained with different agarose concentrations. (**f**) Cross-sectional scanning electron microscopy (SEM) images of freeze-dried 0.3, 0.5, and 1.0 wt% agarose hydrogels, showing concentration-dependent differences in the morphology of the dried networks. Scale bars, 200 μm. (**g**) Radar-plot comparison of the sensitivity, anti-interference performance, and blank-response standard deviation. Each axis retains its original unit and uses an independent scale. The blank-response standard-deviation axis is reversed because a smaller value indicates better stability. (**h**) Transconductance curves of the OECT measured with the hydrogel and liquid electrolytes. (**i**) Output characteristics of the hydrogel-gated OECT measured at different gate voltages, showing typical depletion-mode behavior.

After establishing the device architecture, gate functionalization, and operating conditions, we investigated the effect of hydrogel concentration on the sensing performance. The hydrophilic agarose network retains an aqueous electrolyte and permits penetration of an applied aqueous solution, as qualitatively visualized using methylene blue (Figure S8). Under directional airflow, aerosol droplets carrying target antigens are delivered to the hydrogel surface, where they spread and merge into the matrix (Figure 2a). Under the tested conditions, the resulting concentration-dependent OECT responses indicate that the spike protein remained accessible to the aptamer probes immobilized on the gate electrode, leading to interfacial charge modulation and an electrical response. In this configuration, the hydrogel plays several roles, including aerosol-capture matrix, ion-conducting electrolyte, and pathway for antigen transport, thereby avoiding conventional condensation and off-device liquid-phase processing steps.

As the agarose concentration influences the network density and molecular transport properties of the hydrogel, its effect on device performance was investigated. As shown in Figure 2b, the OECT exhibited a clear concentration-dependent response to the spike protein in 0.5 wt% agarose. Comparison among different agarose concentrations further showed that the device achieved the highest response at 0.5 wt% agarose (Figure 2c and Figure S9), suggesting that this concentration provides a favorable balance for sensing. To evaluate the resistance of the hydrogel-gated OECT to matrix-induced signal perturbation, artificial saliva was used as a representative complex matrix, in which mucin is a major macromolecular component (Figure 2d). The artificial-saliva-induced signal change decreased as the agarose concentration increased, indicating improved resistance to the tested matrix. Previous work has demonstrated that molecular transport through agarose is both size- and concentration-dependent, with higher agarose concentrations imposing greater transport resistance on large biological components [33]. Consistent with this principle, the improved resistance to artificial-saliva-induced perturbation but reduced spike protein response observed at 1.0 wt% agarose suggests that increasing the agarose concentration restricts matrix-component transport while also limiting target accessibility.

Increasing the agarose concentration also increased the variability of the blank response (Figure 2e). The larger blank-response standard deviation observed at 1.0 wt% agarose indicates reduced signal stability. Although the underlying cause was not independently resolved, changes in the hydrogel network may affect ionic coupling and the stability of the hydrogel-gated interface. Thus, improved resistance to artificial-saliva-induced signal perturbation at higher agarose concentrations can coexist with poorer electrical stability because these two performance parameters reflect different aspects of the hydrogel–sensor interface.

SEM images of the freeze-dried hydrogels show qualitative concentration-dependent differences in the apparent network morphology (Figure 2f). The apparent dried network became denser as the agarose concentration increased. A radar plot was used to compare the sensitivity, anti-interference performance, and blank-response standard deviation under different agarose conditions using independent axes that retained their original units (Figure 2g). Although 1.0 wt% agarose provided better anti-interference performance, it showed reduced sensitivity and increased blank-response variability. In contrast, 0.5 wt% agarose produced the highest sensitivity while maintaining acceptable blank stability and anti-interference performance and was therefore selected for subsequent experiments.

To verify that the optimized hydrogel preserved normal transistor operation, the transfer and output characteristics were compared with those obtained using a conventional liquid electrolyte. Compared with the liquid electrolyte, the device with 0.5 wt% agarose hydrogel showed a lower threshold voltage (Figure S10a) and higher transconductance (Figure 2h), indicating more effective electrochemical modulation of the channel. This may be due to better ion accessibility and stronger coupling between the electrolyte and the channel, which can increase the effective volumetric capacitance. The shift in the peak transconductance to higher gate voltages may also indicate a change in capacitive coupling, meaning that a higher gate bias is needed to achieve maximum channel modulation. In addition, the device showed typical depletion-mode output characteristics under both 0.5 wt% agarose hydrogel (Figure 2i) and buffer conditions (Figure S10b).

The device also maintained a similar transfer characteristic when measured in the bent state (Figure S11), supporting its operation under mechanical deformation. After aptamer functionalization and hydrogel coating, the transfer curves remained nearly unchanged during 1 h of exposure to ambient air (Figure S12a). During sealed storage at 4 °C, only modest changes in the transfer curves and transconductance profiles were observed over 3 days (Figure S12b,c), indicating short-term electrical stability under these storage conditions.

### 2.3. Electric-Field-Assisted Sensing Performance

**Figure 3:**
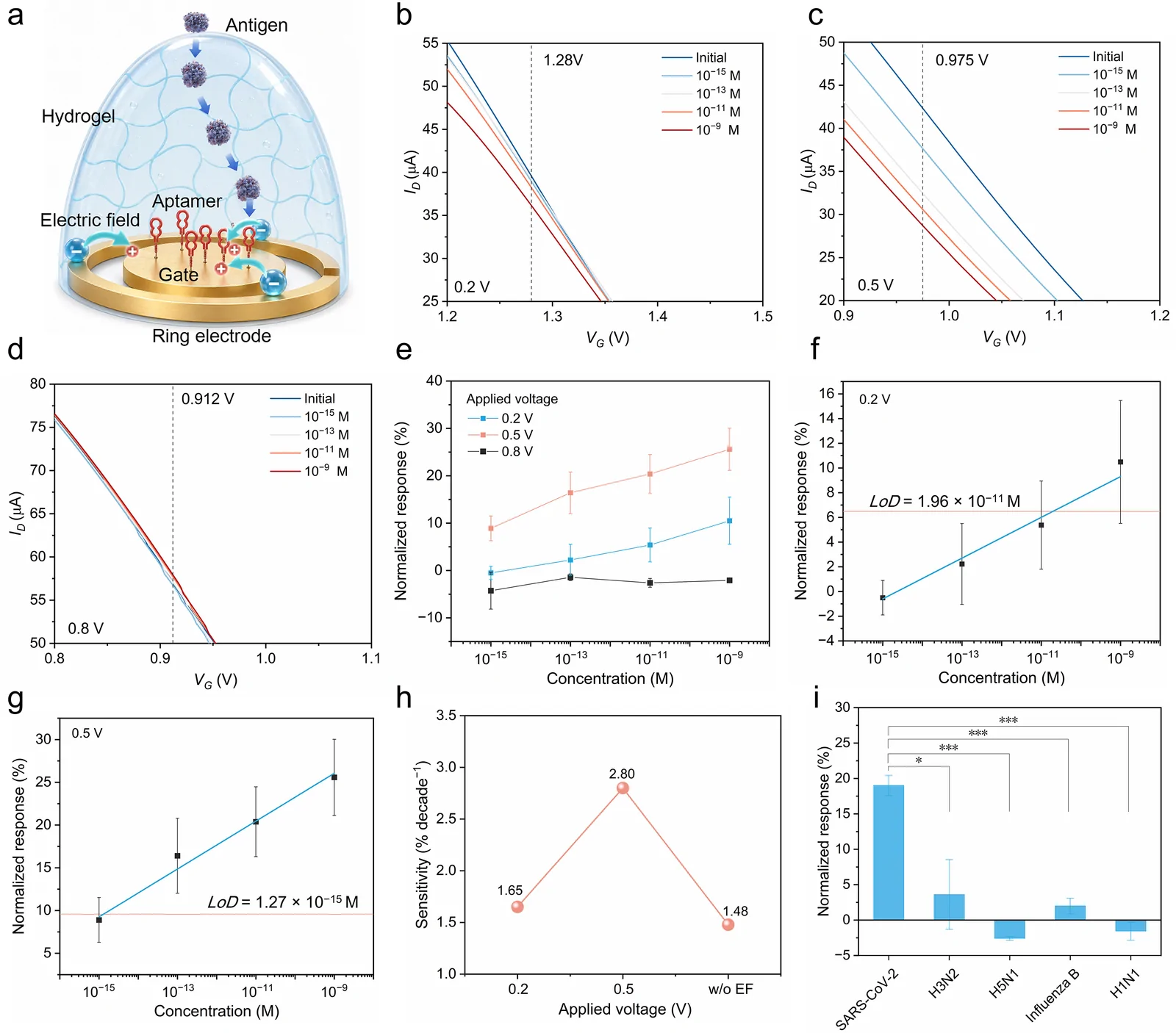
Effect of the electric-field-assistance voltage on the sensing performance of the hydrogel-gated OECT. (**a**) Schematic of the separate 1-min field-assistance step performed before OECT readout. During this step, a positive potential was applied to the aptamer-functionalized Au gate, the ring electrode was grounded, and the source and drain electrodes were electrically floating. Representative transfer characteristics measured after field-assistance steps of 0.2 V (**b**), 0.5 V (**c**), and 0.8 V (**d**) at different SARS-CoV-2 spike protein concentrations. (**e**) Concentration-dependent normalized responses obtained at field-assistance voltages of 0.2, 0.5, and 0.8 V. Calibration curves obtained at 0.2 V (**f**) and 0.5 V (**g**) (*n* = 3; mean ± SE). The red horizontal line represents the detection threshold, defined as the mean blank response plus three times its standard deviation, its intersection with the fitted calibration curve was used to determine the LoD. (**h**) Comparison of calibration sensitivity under no-field, 0.2 V, and 0.5 V conditions. (**i**) Specificity of the sensor using the 0.5 V field-assistance protocol (*n* = 3; mean ± SE). Brackets indicate comparisons with SARS-CoV-2 spike protein (* *p* < 0.05; *** *p* < 0.001, two-sided unpaired *t*-test).

To investigate the effect of electric-field assistance on sensing performance, the ring electrode was positioned around the gate to generate a lateral electric field (Figure 3a). A separate 1-min field-assistance step was applied before OECT readout. This configuration was designed to facilitate target transport through the hydrogel toward the aptamer-functionalized gate, and its effect was evaluated by comparing the concentration-dependent responses obtained under different field-assistance voltages.

The sensing response showed a non-monotonic dependence on the field-assistance voltage applied between the Au gate and the grounded ring electrode (Figure 3b–e). For each device, the response was calculated at the gate voltage corresponding to the maximum transconductance of its baseline transfer curve, and the same gate voltage was used for all subsequent measurements of that device. Increasing the voltage from 0.2 to 0.5 V markedly enhanced the response, whereas a further increase to 0.8 V reduced the response. This result indicates that field-assisted transport requires an optimized operating voltage. Previous work has shown that electric fields can alter the orientation and surface proximity of negatively charged DNA aptamer constructs, thereby affecting target recognition and signal transduction [13]. In the present device, an excessive voltage may reduce the accessibility of the surface-bound aptamer during target capture and perturb the local ion distribution and interfacial gating. These effects may partially offset the enhanced antigen transport at 0.8 V. Quantitative sensing performance was therefore evaluated at 0.2 and 0.5 V. At 0.2 V, the calibration relationship was y = 0.2959 + 0.0197 log_10_*C* (*R*^2^ = 0.982), where y is the normalized response and *C* is the target concentration in molarity (Figure 3f). Using the mean blank response plus three times its standard deviation as the detection threshold, the calculated LoD was 1.96 × 10^−11^ M.

At 0.5 V, the device exhibited a steeper concentration-dependent response (Figure 3e). The transfer curves shifted systematically with increasing SARS-CoV-2 spike protein concentration (Figure S13a), whereas repeated blank-buffer additions produced only minor changes, supporting a stable baseline (Figure S13b). The corresponding calibration curve was fitted as: y = 0.5126 + 0.0280 log_10_*C* (*R*^2^ = 0.988). The fitted calibration yielded a LoD of 1.27 × 10^−15^ M (Figure 3g). The 0.5 V condition showed the best analytical performance among the tested field-assistance voltages. As summarized in Figure 3h, the calibration sensitivity increased from 1.48% decade^−1^ without field assistance and 1.65% decade^−1^ at 0.2 V to 2.80% decade^−1^ at 0.5 V. In addition, the LoD decreased from 1.96 × 10^−11^ M at 0.2 V to 1.27 × 10^−15^ M at 0.5 V (Figure 3f,g). Among the tested conditions, the separate 0.5 V field-assistance step produced the highest concentration-dependent response and was therefore selected for subsequent experiments.

Under the optimized 0.5 V field-assistance protocol, the sensor response to SARS-CoV-2 spike protein was compared with those to four nontarget antigens at the same concentration of 10^−11^ M (Figure 3i). The mean response to spike protein was 19.01%, approximately 5.3 times the 3.60% mean response to H3N2, which had the highest positive mean response among the tested nontarget antigens. These results support the molecular selectivity of the aptamer-functionalized gate under the tested conditions. To assess nonspecific interfacial effects, we compared the aptamer-functionalized gate with a non-functionalized Au gate and a gate modified with non-specific DNA. Upon exposure to spike protein, the non-functionalized gate showed a transfer-curve shift in the direction opposite to that of the aptamer-functionalized gate (Figure S14a,c). The non-specific-DNA-functionalized gate exhibited only a weak response (Figure S14b,c), which may reflect suppression of nonspecific adsorption by the MCH passivation. Together, these controls support specific recognition of spike protein by the aptamer as the origin of the shift toward lower gate voltages.

### 2.4. System Integration and Application Scenarios of the Portable and Wearable Bioelectronic Platform

**Figure 4:**
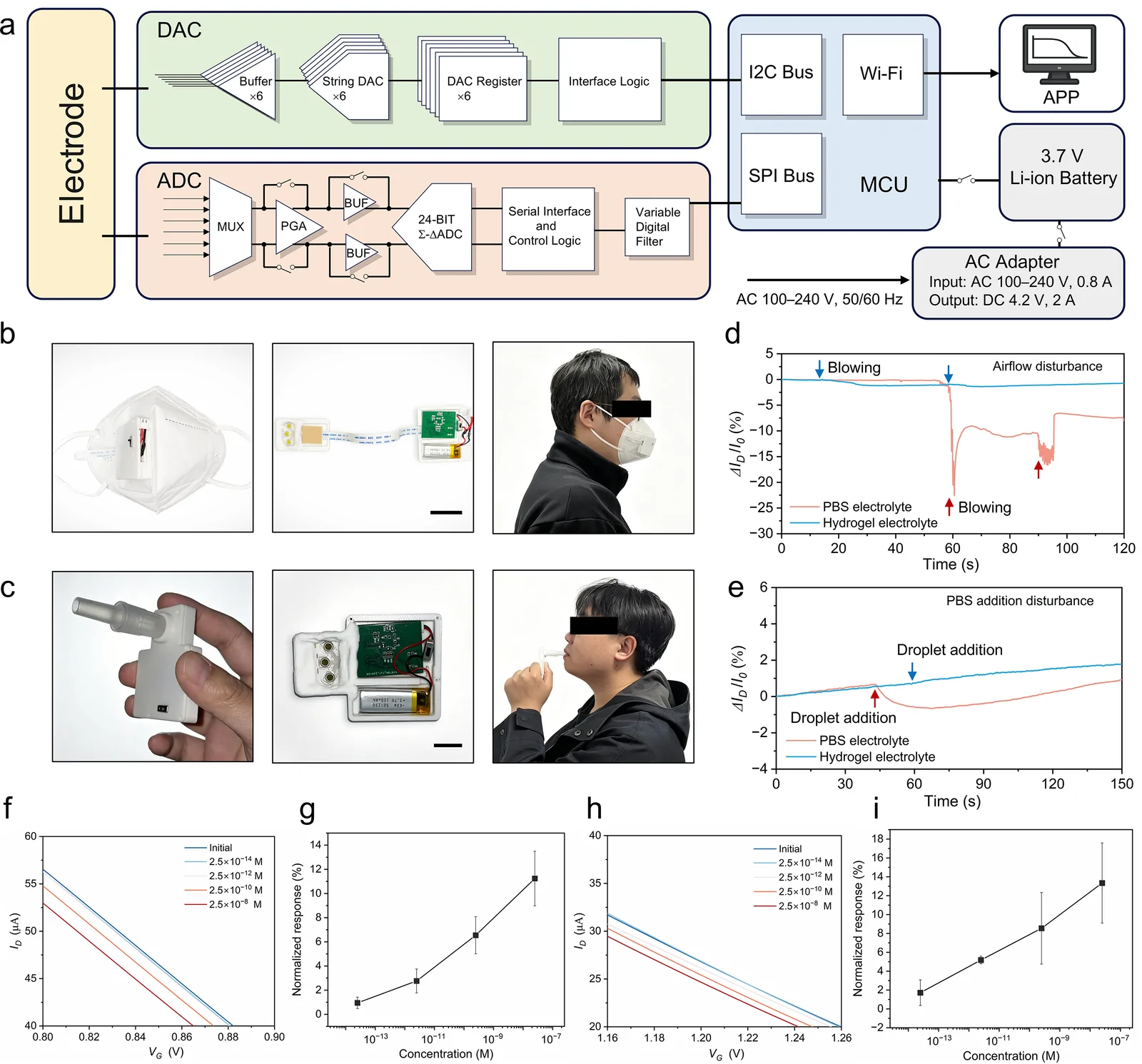
System architecture, disturbance resistance, and dual-mode aerosol sensing with the portable hydrogel-gated OECT platform. (**a**) Hardware architecture of the portable readout system, showing the electrode module, digital-to-analog converter (DAC), analog-to-digital converter (ADC), microcontroller unit (MCU), wireless communication module, power supply, and terminal application. (**b**) Wearable face-mask configuration designed for passive aerosol collection during a single wearing period. Scale bar, 2 cm. (**c**) Handheld configuration for active aerosol sampling through a directed mouthpiece. Scale bar, 1 cm. Responses of OECTs using hydrogel or phosphate buffered saline (PBS) electrolytes to airflow disturbance (**d**) and droplet addition disturbance (**e**). Face-mask configuration: representative transfer characteristics (**f**) and concentration-dependent normalized response (**g**) (*n* = 3; mean ± SE). Handheld configuration: representative transfer characteristics (**h**) and concentration-dependent normalized response (**i**) (*n* = 3; mean ± SE).

To support portable operation, wireless data transmission, and system-level integration, a compact bioelectronic readout platform was developed for the hydrogel-gated OECT sensor. As shown in Figure 4a, the system comprises an electrode interface, a digital-to-analog converter (DAC), an analog-to-digital converter (ADC), a microcontroller unit (MCU), a wireless communication module, a terminal application (app), and a power supply. The MCU serves as the central controller and communicates with the DAC and ADC through I2C and SPI buses, respectively, enabling programmable voltage output and integrated electrical signal acquisition.

The detailed circuit schematic of the readout system is shown in Figure S15. The system comprises an ESP8266-based MCU, an AD5675-based programmable DAC, an AD7124-based high-resolution ADC, power-management and peripheral circuits, and interfaces for the hydrogel-gated OECT sensor. The DAC supplies programmable drain and gate bias voltages for OECT operation and controls the voltage used to generate an electric field within the hydrogel. The ADC digitizes the voltage output of the sensing circuit, from which changes in the OECT drain current are determined. The MCU subsequently processes the acquired data and wirelessly transmits them to a terminal application for real-time visualization. All electronic components were integrated onto a compact PCB, powered by a 3.7 V rechargeable lithium-ion battery, and enclosed in a 3D-printed casing. When fully charged, the assembled system can operate continuously for approximately 1.5 h, which is sufficient for short-term portable sensing measurements.

To demonstrate the applicability of the proposed platform in different scenarios, two sensing configurations were developed: a wearable face-mask configuration and a handheld configuration. In the wearable configuration, the sensor was integrated into a face mask for passive aerosol collection during a single wearing period (Figure 4b). This configuration is intended to allow aerosol sampling before one electrical readout; each sensing electrode is used for one sampling and analysis cycle and then discarded. In the handheld configuration, the sensor and readout electronics were integrated into a compact device with a mouthpiece that directs a controlled aerosol stream toward the sensing interface (Figure 4c). This configuration prioritizes rapid, user-initiated measurements and is better suited to spot testing, triage, or point-of-care screening. The two configurations therefore address distinct use scenarios: the face mask emphasizes passive aerosol collection over a single sampling period, whereas the handheld device emphasizes short-duration, directed sampling.

The hydrogel electrolyte also enhanced the operational robustness of the sensing interface. Under direct airflow, the hydrogel-gated device exhibited only a small transient response, whereas the liquid-electrolyte device showed a pronounced signal excursion (Figure 4d). A similar trend was observed during droplet addition, with the hydrogel interface suppressing abrupt perturbations relative to the phosphate buffered saline (PBS) electrolyte (Figure 4e). These results indicate that the gel matrix stabilizes the electrolyte geometry and mitigates airflow- and liquid-addition-induced fluctuations, which is advantageous for portable aerosol sampling.

Aerosolized target samples were generated by nebulizing SARS-CoV-2 spike protein solutions and delivered to the face-mask and handheld devices using the setups shown in Figure S16a,b. The medical nebulizer produces aerosol particles with a diameter below 5 μm, comparable to the size range of aerosols naturally generated during human respiration. Throughout the aerosol experiments, the reported experimental concentrations refer to the protein concentrations in the source solutions loaded into the nebulizer rather than directly measured airborne concentrations. The face-mask configuration exhibited systematic transfer-curve shifts with increasing target concentration (Figure 4f) and a corresponding concentration-dependent normalized response (Figure 4g). The fitted calibration curve yielded an LoD of 6.89 × 10^−12^ M expressed in terms of the nebulizer source-solution concentration (Figure S17a). The handheld configuration likewise exhibited systematic transfer-curve shifts and a concentration-dependent normalized response (Figure 4h,i). Its fitted calibration curve yielded an LoD of 1.19 × 10^−12^ M expressed in terms of the nebulizer source-solution concentration (Figure S17b). For comparison with the mass-balance approach reported by Li et al. [24], the source-solution detection thresholds were also converted using the literature-based ratio between the estimated liquid atomization rate and the carrier-air flow rate. Under this idealized model, the source-solution thresholds correspond to theoretical aerosol-equivalent concentration estimates of 4.92 × 10^−16^ M and 8.50 × 10^−17^ M of carrier air for the face-mask and handheld configurations, respectively. In contrast, the PBS aerosol control produced negligible variation (Figure S18), indicating that the measured response originated predominantly from the aerosolized target rather than from nebulization or buffer exposure. Across matched target concentrations, the handheld configuration produced larger responses than the face-mask configuration. This enhancement may be attributed to the mouthpiece-guided flow path of the handheld device, which confines and directs the incoming aerosol stream toward the hydrogel sensing interface, thereby likely increasing target–interface contact while reducing aerosol dispersion within the sampling space relative to the more open face-mask geometry. In additional aerosol tests, nebulized artificial saliva containing spike protein at source concentrations of 10^−9^ and 10^−7^ M produced larger responses than antigen-free artificial saliva (Figure S19), supporting target detection in an aerosolized complex matrix. Table 1 compares representative respiratory aerosol pathogen detection approaches in terms of detection targets, LoD concentration bases, sample pretreatment, and reported sampling-to-result times. 

## 3. Conclusions

In summary, we developed a hydrogel-gated OECT platform for the direct electrical detection of aerosol-borne viral antigens. The non-flowing agarose hydrogel replaced the conventional liquid electrolyte and integrated aerosol capture, ionic coupling, and antigen transport within a single sensing interface, while reducing airflow- and droplet-induced signal perturbations. The ring electrode enabled a separate 1-min electric-field-assistance step before OECT readout, and the highest concentration-dependent sensing response was obtained at 0.5 V. Under the optimized conditions, the platform achieved a solution-phase LoD of 1.27 fM and a sample-to-result time of 17 min, while retaining selective responses under the tested interference conditions. Integration with a portable wireless readout system enabled implementation in both face-mask and handheld configurations.

For nebulized SARS-CoV-2 spike protein, the calibration-based LoDs expressed in terms of the nebulizer source-solution concentrations were 6.89 × 10^−12^ M and 1.19 × 10^−12^ M for the face-mask and handheld configurations, respectively. Collectively, this platform integrates aerosol capture, ring-electrode field assistance, aptamer-mediated recognition, and OECT signal transduction without prior condensation, elution, or off-device liquid-phase processing. These findings support the feasibility of aerosol-to-electrical transduction for wearable and portable detection of aerosol-borne viral antigens.

The present aerosol experiments used nebulized recombinant SARS-CoV-2 spike protein under controlled conditions. Although short-term electrical stability and resistance to individual airflow disturbances were evaluated, sensing performance under repeated breathing-like airflow, varying ambient humidity, and prolonged environmental exposure remains to be established. Future studies should evaluate clinical exhaled-breath samples and intact respiratory pathogens against appropriate molecular reference assays to determine diagnostic performance and examine how direct aerosol antigen detection may complement rapid point-of-care molecular methods, including ultrafast multiplex PCR [40].

## 4. Materials and Methods

### 4.1. Electrode Fabrication

A 188 μm-thick polyethylene terephthalate (PET) substrate was cut to the required dimensions and sequentially cleaned in acetone, ethanol, isopropyl alcohol (IPA), and deionized (DI) water for 10 min each, followed by drying under nitrogen. The substrate was then treated with oxygen plasma at 100 W for 150 s using a VP-R series plasma cleaner (Guangzhou SunJune Technology Co., Ltd., Guangzhou, China). BP212-37S photoresist (RAE) was spin-coated at 500 rpm for 10 s and 5000 rpm for 50 s, followed by prebaking at 100 °C for 2 min. After ultraviolet exposure for 10 s, the substrate was post-baked at 100 °C for 1 min and developed using KMP PD238-II developer. Cr and Au layers were sequentially deposited by thermal evaporation using a thermal evaporator (DM300, Hefei Jusheng Vacuum Technology Co., Ltd., Hefei, China) at a deposition rate of 0.5 Å s^−1^ to thicknesses of 10 and 100 nm, respectively. Lift-off was performed by immersing the samples in acetone overnight. The resulting electrodes were sequentially cleaned with acetone, ethanol, IPA, and DI water and dried under nitrogen.

### 4.2. PEDOT:PSS Channel Patterning

PEDOT:PSS (Clevios PH1000) was modified with ethylene glycol (EG), dodecylbenzenesulfonic acid (DBSA), and (3-glycidyloxypropyl)trimethoxysilane (GOPS). Briefly, 5.0 g of EG was mixed with 0.1 g of DBSA. A 51 mg aliquot of this mixture was combined with 10 mg of GOPS and 939 mg of PH1000 and vortexed until homogeneous. BP212-37S photoresist and KMP PD238-II developer were used to define the PEDOT:PSS channel pattern. Before deposition of the modified PEDOT:PSS, the patterned substrate was treated with oxygen plasma at 100 W for 60 s. The modified PEDOT:PSS dispersion was spin-coated at 3000 rpm for 60 s and thermally annealed at 90 °C for 30 min. Lift-off was performed by immersing the samples in acetone for 10 min twice, followed by ultrasonication in acetone for 30 s. The devices were then rinsed with DI water and dried under nitrogen. The resulting PEDOT:PSS channels had a width of 1000 μm and a length of 70 μm.

### 4.3. Au Gate Functionalization

A thiolated DNA aptamer with the sequence 5′-SH-C6-TTTTTTTTTTCAGCACCGACCTTGTGCTTTGGGAGTGCTGGTCCAAGGGCGTTAATGGACA-3′ was synthesized by GenScript (Nanjing, China) and purified by high-performance liquid chromatography (HPLC) The 51-nucleotide recognition sequence corresponds to the CoV2-RBD-1C aptamer reported by Song et al.; the 5′ thiol and poly(T) spacer were added for immobilization on the Au gate [41]. The aptamer was diluted to 1 μM in 1× PBS (pH 7.4) and incubated with 100 μM tris(2-carboxyethyl)phosphine (TCEP) for 1 h in the dark to reduce the terminal disulfide bonds. The solution was subsequently heated at 90 °C for 3 min and gradually cooled to room temperature to promote aptamer folding. MCH was then added to the solution at a final concentration of 9 μM.

Before functionalization, the Au gate electrodes were treated with oxygen plasma at 100 W for 20 s. The aptamer/MCH solution was applied to the Au gates and incubated for 16 h at room temperature under humidified and dark conditions. The electrodes were subsequently immersed in 1× PBS for 10 min and treated with 1 mM MCH in 1× PBS for 1 h to passivate the remaining exposed Au surface. The electrodes were rinsed twice with 1× PBS for 5 min and twice with 0.1× PBS containing 2 mM anhydrous MgCl_2_ for 5 min. The functionalized electrodes were stored at 4 °C while covered with 0.1× PBS containing 2 mM MgCl_2_ and used within 3 days.

### 4.4. Electrochemical Characterization

Electrochemical characterization was performed using a CHI600E electrochemical workstation (Shanghai Chenhua Instrument Co., Ltd., Shanghai, China) in a conventional three-electrode configuration, with the Au gate electrode as the working electrode, a Pt wire as the counter electrode, and an Ag/AgCl electrode as the reference electrode. Measurements were conducted in 1× PBS (pH 7.4) containing 5 mM K_3_[Fe(CN)_6_] and 5 mM K_4_[Fe(CN)_6_]·3H_2_O. Cyclic voltammetry was performed from −0.5 to 0.8 V versus Ag/AgCl at a scan rate of 50 mV s^−1^. Ten consecutive cycles were recorded, and the final cycle was used for analysis. Electrochemical impedance spectroscopy was performed at the open-circuit potential over a frequency range of 0.1 Hz to 100 kHz using an AC perturbation amplitude of 5 mV. The impedance spectra were fitted using a Randles equivalent circuit.

### 4.5. Agarose Hydrogel Preparation and Device Assembly

Agarose hydrogels with concentrations of 0.3, 0.5, and 1.0 wt% were prepared in 0.1× PBS (pH 7.4) containing 2 mM MgCl_2_. The mixtures were heated to 95 °C in a water bath (Tianjin Saidelisi Experimental Analytical Instrument Factory, Tianjin, China) under continuous stirring until the agarose was completely dissolved and were subsequently maintained at 60 °C for 30 min. The devices were immersed in and withdrawn from the agarose solution three consecutive times to coat the active sensing region. The deposited agarose was allowed to undergo initial gelation at room temperature, followed by further gelation at 4 °C for 30 min. The hydrogel-coated devices were stored at 4 °C in 0.1× PBS containing 2 mM MgCl_2_ and used within 3 days.

### 4.6. SEM Characterization of Agarose Hydrogels

Agarose hydrogels with concentrations of 0.3, 0.5, and 1.0 wt% were frozen at −80 °C for 1 h. The freeze dryer (SCIENTZ-10N, Ningbo Scientz Biotechnology Co., Ltd., Ningbo, China) was precooled to −40 °C for 40 min, after which the samples were dried under vacuum for 7–8 h. The freeze-dried samples were immersed in liquid nitrogen and fractured to expose their cross-sectional surfaces. The samples were then mounted on SEM stubs, sputter-coated with gold, and examined using a ZEISS Sigma 300 scanning electron microscope (Carl Zeiss Microscopy GmbH, Jena, Germany) in secondary-electron imaging mode at an accelerating voltage of 3.0 kV. Images were acquired at different magnifications, with the corresponding scale bars indicated in the images. Because freezing, freeze-drying, and cryogenic fracturing may alter the native hydrogel network, the SEM images were used only for qualitative comparison of the cross-sectional morphologies of the freeze-dried hydrogels and not for quantitative determination of the pore dimensions of the hydrated gels.

### 4.7. OECT Electrical Characterization

The electrical characteristics of the OECTs were measured using a Keithley 2612B source meter (Keithley Instruments, Inc., Solon, OH, USA). Output characteristics were recorded by sweeping the drain voltage (*V_D_*) from 0 to 0.15 V in 0.001 V increments while varying the gate voltage (*V_G_*) from 0 to 1.2 V in 0.2 V steps. During transfer-characteristic readout, the ring electrode was disconnected from all power supplies and measurement instruments, the drain voltage (*V_D_*) was fixed at 0.04 V, and the gate voltage (*V_G_*) was swept from 0 to 1.5 V in 0.001 V increments. Six consecutive transfer scans were recorded for each measurement to obtain a stable device response. The six scans required approximately 1 min in total, and the sixth scan was used for subsequent data analysis.

The transconductance magnitude was calculated as g_m_ = |∂*I_D_*/∂*V_G_*|. The *V_G_* corresponding to the maximum transconductance of the baseline transfer curve was selected as the analysis voltage. The drain current at this fixed *V_G_* was extracted from all subsequent transfer curves. The normalized response was calculated as: −Δ*I*/*I*_0_ = (*I*_0_ − *I*)/*I*_0_. Where *I*_0_ and *I* represent the drain currents measured before and after target exposure, respectively. Unless otherwise stated, quantitative data are presented as the mean ± standard error (SE) obtained from three independent devices (*n* = 3).

### 4.8. Solution-Phase Antigen Detection

SARS-CoV-2 spike S1+S2 extracellular domain recombinant protein bearing a His tag (catalog no. 40589-V08B1, Sino Biological, Inc., Beijing, China) was serially diluted in 0.1× PBS (pH 7.4) containing 2 mM MgCl_2_ to obtain antigen concentrations ranging from 10^−18^ to 10^−8^ M. Before target addition, six consecutive baseline transfer scans were recorded for each device, and the sixth scan was used as the baseline transfer characteristic. For the comparison of different agarose concentrations under no-field conditions, devices incorporating 0.3, 0.5, or 1.0 wt% agarose hydrogel were evaluated. After baseline measurement, 2 μL of the antigen solution was applied to the hydrogel sensing interface and incubated for 20 min without electric-field assistance. Six consecutive transfer scans were then recorded without washing the hydrogel, and the sixth scan was used for data analysis.

For the field-assisted measurements, devices incorporating the selected 0.5 wt% agarose hydrogel were used. After baseline measurement, 2 μL of the antigen solution was applied to the hydrogel sensing interface and allowed to stand for 10 min. A separate 1-min electric-field-assistance step was subsequently performed, during which the ring electrode was grounded, +0.2, +0.5, or +0.8 V was applied to the aptamer-functionalized Au gate, and the source and drain electrodes were left electrically floating. No drain current was recorded during this step. The applied voltage was then removed, and the ring electrode was disconnected from all power supplies and measurement instruments. Six consecutive transfer scans were subsequently recorded without washing the hydrogel, and the sixth scan was used for data analysis. The overall sample-to-result time was approximately 17 min, comprising a 10-min sample-exposure and incubation period, a 1-min electric-field-assistance step, a subsequent 5-min incubation, and approximately 1 min for electrical readout.

Artificial-saliva interference analysis was performed separately under no-field conditions. Commercially available artificial saliva without added target antigen was used (Saliva juice, pH 6.8; catalog no. R41109, Shanghai Yuanye Bio-Technology Co., Ltd., Shanghai, China). According to the manufacturer, the formulation contains 25 mg L-1 mucin together with NaCl, KCl, potassium thiocyanate, sodium sulfate, NaH2PO4, urea, sodium bicarbonate, amylase, and uric acid. Devices incorporating 0.3, 0.5, or 1.0 wt% agarose hydrogel were evaluated. After baseline measurement using the same six-scan procedure, 2 μL of artificial saliva was applied to the hydrogel surface and incubated for 20 min without electric-field assistance. Six consecutive transfer scans were then recorded without washing the hydrogel, and the sixth scan was used for data analysis. The artificial-saliva-induced relative signal change was calculated as *R*_int_ = |*I*_AS_ − *I*_0_|/|*I*_0_|, where *I*_0_ and *I*_AS_ represent the drain currents measured before and after exposure to artificial saliva, respectively, at the same fixed analysis gate voltage. The anti-interference performance was calculated as *P*_AI_ = (1 − *R*_int_) × 100%, where a larger *P*_AI_ value indicates a smaller artificial-saliva-induced signal perturbation and therefore better resistance to the tested matrix.

### 4.9. Limit of Detection and Data Analysis

Blank measurements were performed using three independent devices (*n* = 3) exposed to 0.1× PBS (pH 7.4) containing 2 mM MgCl_2_. The response threshold for determining the limit of detection was defined as *μ* + 3*σ*, where *μ* and *σ* are the mean and standard deviation of the blank responses, respectively. A linear calibration relationship was established between the normalized response and the logarithm of the antigen concentration: y = a + b log_10_*C*, where a and b are the intercept and slope of the calibration curve, respectively. The LoD was calculated as:

LoD = 10 ^(^*^μ^*
^+ 3^*^σ^*
^− a)/b^

### 4.10. Aerosol Generation and Detection

SARS-CoV-2 Spike S1+S2 protein was diluted in 0.1× PBS (pH 7.4) containing 2 mM MgCl_2_ to prepare nebulization source solutions at concentrations of 2.5 × 10^−14^ to 2.5 × 10^−8^ M. For each detection experiment, 2 mL of the source solution was loaded into a medical nebulizer (NE-C28, OMRON Dalian Co., Ltd., Dalian, China). The face-mask configuration was exposed to the generated aerosol for 5 min, whereas aerosol was delivered to the handheld configuration for 2 min. The devices were then allowed to stand for an additional 5 and 8 min, respectively, so that the combined aerosol-exposure and standing period was 10 min for both configurations. After aerosol exposure, the same sequential field-assistance and OECT-readout protocol described for solution-phase detection was used. Briefly, the ring electrode was grounded, +0.5 V was applied to the aptamer-functionalized Au gate for 1 min, and the source and drain electrodes were electrically floating. The voltage was then removed, the ring electrode was disconnected, and the OECT transfer characteristics were recorded after an additional 5-min incubation. Blank aerosol controls were generated by nebulizing the same antigen-free buffer. The overall sample-to-result time was approximately 17 min, including a combined 10-min aerosol-exposure and standing period, a 1-min electric-field-assistance step, a subsequent 5-min incubation, and approximately 1 min for electrical readout. The reported source concentrations refer to the protein concentrations in the solutions loaded into the nebulizer.

Following the mass-balance approach reported by Li et al. [24], the aerosol-equivalent concentration was theoretically estimated from the ratio of the liquid atomization rate to the airflow rate. An NE-C28 nebulizer operating at 3.5 L min^−1^ was reported to atomize approximately 2.5 mL of solution within 10 min, corresponding to a liquid atomization rate of:

Qliq,ref=2.5 mL10 min=0.25 mL min−1.

The theoretical conversion factor was therefore:

Fref=Qliq,refQair=0.25 mL min−13.5 L min−1=7.14×10−5LsolutionLair−1.

The aerosol-equivalent concentration was calculated as: *C_aerosol_*_,_*_eq_* = F_ref_ *C_source_*. Accordingly, the source-solution range corresponded to the following theoretical aerosol-equivalent range: 2.5 × 10^−14^ − 2.5 × 10^−8^ M → 1.785 × 10^−18^ − 1.785 × 10^−12^ M. These values are idealized mass-balance estimates and do not represent directly measured airborne concentrations or experimentally validated airborne LoDs.

### 4.11. Selectivity Tests

Sensor selectivity was evaluated using SARS-CoV-2 Spike S1+S2 recombinant protein as the target and recombinant H3N2, H5N1, influenza B, and H1N1 antigens as nontarget controls. Each antigen was tested separately at 10^−11^ M. For each measurement, 2 μL of the corresponding antigen solution was applied to the hydrogel sensing interface. The device was allowed to stand for 10 min and was then subjected to the same separate 1-min field-assistance step, with the ring electrode grounded, +0.5 V applied to the Au gate, and the source and drain electrodes electrically floating. After removal of the voltage and disconnection of the ring electrode, the OECT transfer characteristic was measured following an additional 5-min incubation. Each condition was evaluated using three independent devices (*n* = 3).

## Figures and Tables

**Table 1:** Comparison of representative respiratory aerosol pathogen detection approaches and their sample-processing requirements.

Study	Detection Target	LoD and Concentration Basis	Sample Pretreatment	Sampling to Result
This work	SARS-CoV-2 spike (S1+S2) protein	6.89 pM (face mask); 1.19 pM (handheld); nebulizer source solution	Direct hydrogel aerosol capture	17 min total (10 min sampling/standing, 1 min field assistance, 5 min incubation, ~1 min readout)
Li et al. [24]	SARS-CoV-2 nucleocapsid protein (NP)	19.7 fg/L (estimated aerosol)	Direct hydrogel aerosol sensing	10 min response after exposure; complete sample-to-result interval not reported
Wang et al. [34]	SARS-CoV-2 Spike; Influenza A hemagglutinin	0.1 fg/mL (gaseous-media test; air-volume basis unverified)	Direct exposure to atomized or carrier-gas samples	10 min response; complete sample-to-result interval not reported
Xue et al. [35]	SARS-CoV-2 Spike; Porcine transmissible gastroenteritis virus (TGEV)	7 PFU/mL (atomized source sample)	Direct collection on mask-mounted nanowire sensor	5 min atomization/detection; complete sample-to-result interval not reported
Nguyen et al. [21]	SARS-CoV-2 RNA	500 copies per reaction (17 aM)	On-mask reagent release, lysis, amplification, and CRISPR readout	30 min collection + ~90 min assay
Daniels et al. [36]	SARS-CoV-2 viral particles (Spike S1 recognition)	10 PFU/mL (in cultured virus suspensions)	Pre-cooled mask; exhaled breath condensate (EBC) to aptasensor	20 min pre-cooling + 5 min breath collection + 10 min sensor incubation; additional measurement time excluded
Dörfler et al. [37]	SARS-CoV-2 RNA (ORF1a/b and E genes)	~10^4^ copies/mL (reported EBC LoD)	Chilled mask collects EBC; liquid condensate is analyzed by off-device RT-qPCR	30 min pre-cooling + 5 min EBC collection; complete sample-to-result interval not reported
Chen et al. [38]	SARS-CoV-2 pseudovirus	1.6 fg/mL (pseudovirus)	On-device EBC condensation	8 min, reported from collection to readout (EBC collection within 1 min)
Leding et al. [39]	SARS-CoV-2 RNA (E gene)	LoD not reported	Electrostatic capture; elution + RT-qPCR	4 min air collection; complete sample-to-result interval not reported
Li et al. [20]	SARS-CoV-2 spike protein, pseudovirus, and live virus	1 fg/mL (Spike); 0.5 pg/mL (pseudovirus); liquid-phase tests	Direct breath/blow capture into an on-device PBS microchannel	110 s blow; 350 s breath; complete sampling-to-result interval not reported

## Data Availability

The data that support the findings of this study are available from the corresponding authors upon reasonable request.

## Supplementary Materials

The following supporting information can be downloaded at https://www.mdpi.com/article/10.3390/gels12100916/s1, Figures S1–S19. Figure S1: Schematic illustration of the electrode structure. Scale bar, 200 μm; Figure S2: Randles equivalent circuit model used to analyze the impedance spectra. The circuit consists of the electrolyte resistance (*R*_s_), the electric double-layer capacitance (*C_dl_*) formed at the electrode/electrolyte interface, the charge transfer resistance (*R_ct_*) of the electrode, and the Warburg impedance (*Z_w_*) resulting from ion diffusion from the electrolyte to the electrode surface; Figure S3: Transfer curves of the OECT in response to SARS-CoV-2 S protein measured in solution electrolyte (0.1× PBS with 2 mM MgCl_2_); Figure S4: SEM images of the gate interface. (a) Bare Au electrode. (b) Gate electrode after functionalization. Scale bar, 200 nm; Figure S5: Transfer curves measured in solution electrolyte (0.1× PBS). (a) Transfer curves obtained after addition of different concentrations of S protein. (b) Transfer curves obtained after four consecutive additions of 0.1× PBS without Mg^2+^; Figure S6: Concentration-dependent sensor responses measured at room temperature and 4 °C (n = 5, mean ± SE); Figure S7: Device stability test. (a) Transfer curves after five consecutive additions of 0.1× PBS. (b) Transfer curves of the non-functionalized gate sensor after addition of S protein; Figure S8: Penetration experiment of solution in the 0.5 wt% agarose hydrogel. Methylene blue solution (0.1%) was dropped onto agarose hydrogel with a thickness of 2 mm; Figure S9: Linear fitting of S protein responses measured with hydrogels of different concentrations. (a) 0.3 wt%. (b) 0.5 wt%. (c) 1 wt%; Figure S10: (a) Comparison of transfer curves measured with hydrogel and 0.1× PBS electrolytes. (b) Output characteristics measured in 0.1× PBS; Figure S11: Comparison of the OECT transfer characteristics measured in the flat and bent states. Photographs show the device in the corresponding measurement configurations; Figure S12: Temporal stability of aptamer-functionalized, hydrogel-coated OECTs. (a) Transfer characteristics measured initially and after 30 and 60 min of exposure to ambient air. (b) Transfer characteristics measured on days 0–3 during sealed storage at 4 °C. (c) Transconductance curves calculated from the transfer characteristics in (b); Figure S13: (a) Transfer curves measured under a 0.5 V electric field. (b) Blank control; Figure S14: Specificity controls for SARS-CoV-2 spike protein detection. Representative OECT transfer characteristics obtained after exposure to increasing concentrations of spike protein using (a) a non-functionalized gate and (b) a gate modified with a non-specific thiolated DNA control (5′-SH-C6-TTTTTTTTTTGGTGCTGGAGAG-3′). (c) Comparison of the normalized responses of the aptamer-functionalized, non-functionalized, and non-specific-DNA-functionalized gates. All devices were evaluated using the same 0.5 V field-assistance and subsequent OECT-readout protocol (n = 3; mean ± SE); Figure S15: Circuit diagram of the portable device; Figure S16: Nebulizer-based simulation test. (a) Face-mask-based test. (b) Handheld test; Figure S17: Fitted calibration curve for concentration-dependent responses under nebulizer-based testing. (a) Face-mask-based test, *R*^2^ = 0.996. (b) Handheld test, *R*^2^ = 0.994. n = 3; mean ± SE; Figure S18: Blank control: nebulization test using 0.1× PBS solution. (a) Face-mask-based test. (b) Handheld test; Figure S19: Detection of SARS-CoV-2 spike antigen in artificial saliva. Representative OECT transfer curves before and after exposure to nebulized artificial saliva containing (a) 10^−9^ M or (b) 10^−7^ M spike antigen, and (c) antigen-free artificial saliva. (d) Normalized responses to antigen-free artificial saliva (AS) and artificial saliva containing spike antigen at the indicated concentrations.

## Abbreviations

The following abbreviations are used in this manuscript:

ADC	Analog-to-digital converter
CV	Cyclic voltammetry
DAC	Digital-to-analog converter
DBSA	Dodecylbenzenesulfonic acid
EG	Ethylene glycol
GOPS	(3-Glycidyloxypropyl)trimethoxysilane
LoD	Limit of detection
MCH	6-Mercapto-1-hexanol
MCU	Microcontroller unit
OECT	Organic electrochemical transistor
PCB	Printed circuit board
PBS	Phosphate buffered saline
PDMS	Polydimethylsiloxane
PEDOT:PSS	Poly(3,4-ethylenedioxythiophene):poly(styrene sulfonate)
PET	Polyethylene terephthalate
SEM	Scanning electron microscopy
TCEP	Tris(2-carboxyethyl)phosphine
EBC	Exhaled breath condensate
TGEV	Transmissible gastroenteritis virus
