# Supplementary material for: Hydrogel-Gated Organic Electrochemical Transistors for Wearable and Portable Direct Detection of Aerosol-Borne Viral Antigens
Source: Gels. 2026 Oct 12;12(10):916. doi: 10.3390/gels12100916 (PMC13704137; doi:10.3390/gels12100916)
Supplement: Supplementary file 1 [file gels-12-00916-s001.zip › gels-4567722-supplementary.pdf]

Supplementary Material

Hydrogel-Gated Organic Electrochemical Transistors for Wearable and Portable Direct Detection of Aerosol-Borne Viral Antigens

Haiyang Yu[1], Yuhan Lu[1], Bihan Guo[1], Jiameng Wang[1], Ya Zhang[1,], Bingfang Wang[1], Qiuyu Yang[1], Lan Shen[1], Mengtian Shan[2], Feilong Wang[2], Daizong Ji[1,*], Yin Fang[1]*

[1]The Institute for Biomedical Engineering & Nano Science, School of Medicine, Tongji University, Shanghai 200331, China

[2]Department of Pulmonary and Critical Care Medicine, Shanghai East Hospital, School of Medicine, Tongji University, Shanghai 200120, China

Correspondence to: *yazhang@tongji.edu.cn (Y.Z.); jidaizong@tongji.edu.cn (D.J.)*

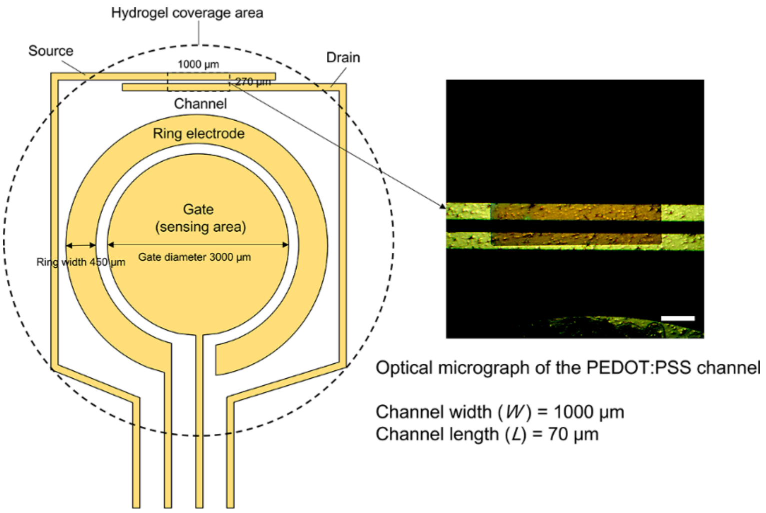

Figure S1. Schematic illustration of the electrode structure. Scale bar, 200 µm.

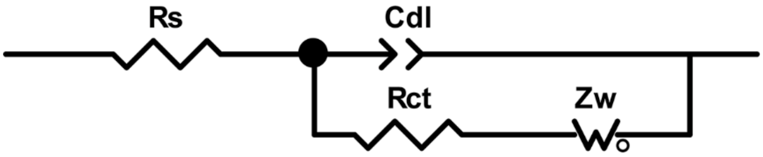

Figure S2. Randles equivalent circuit model used to analyze the impedance spectra. The circuit consists of the electrolyte resistance (R_s), the electric double-layer capacitance (C_{dl}) formed at the electrode/electrolyte interface, the charge transfer resistance (R_{ct}) of the electrode, and the Warburg impedance (Z_w) resulting from ion diffusion from the electrolyte to the electrode surface.

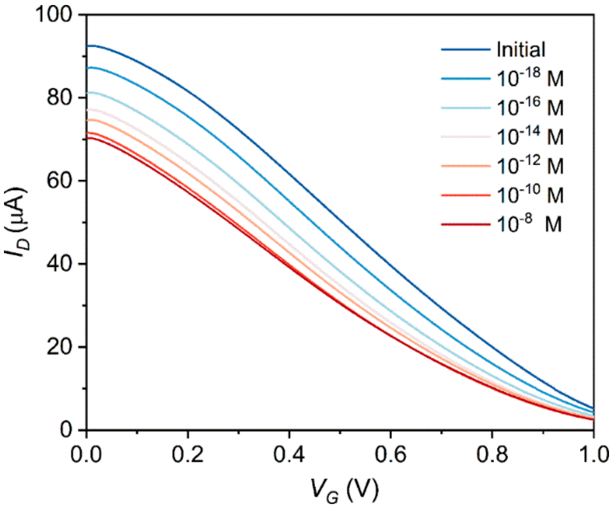

Figure S3. Transfer curves of the OECT in response to SARS-CoV-2 S protein measured in solution electrolyte (0.1× PBS with 2 mM $MgCl_2$).

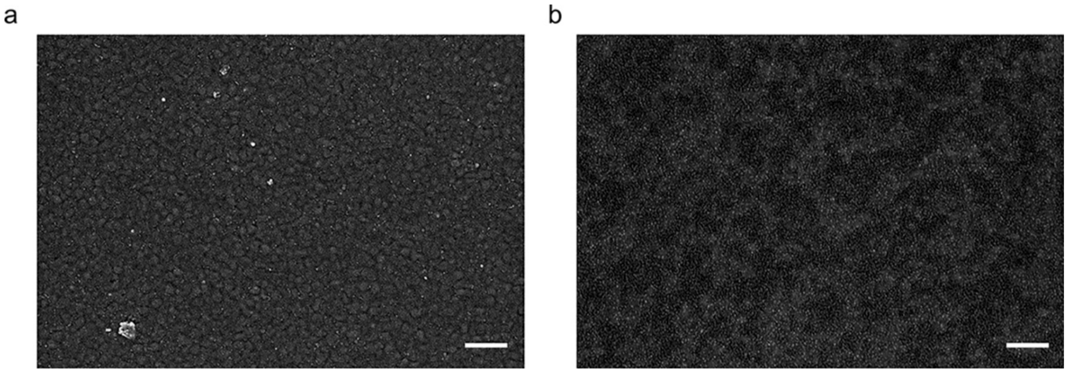

Figure S4. SEM images of the gate interface. (a) Bare Au electrode. (b) Gate electrode after functionalization. Scale bar, 200 nm.

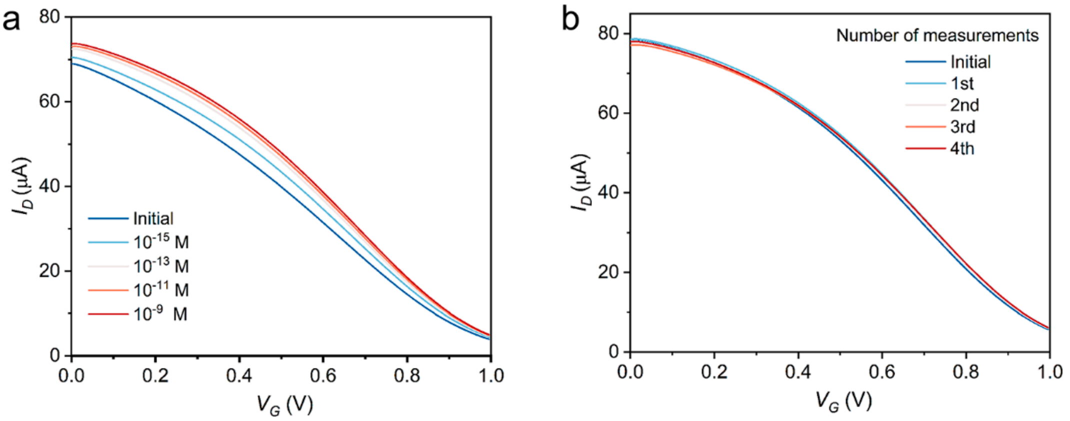

Figure S5. Transfer curves measured in solution electrolyte (0.1× PBS). (a) Transfer curves obtained after addition of different concentrations of S protein. (b) Transfer curves obtained after four consecutive additions of 0.1× PBS without Mg^{2+}.

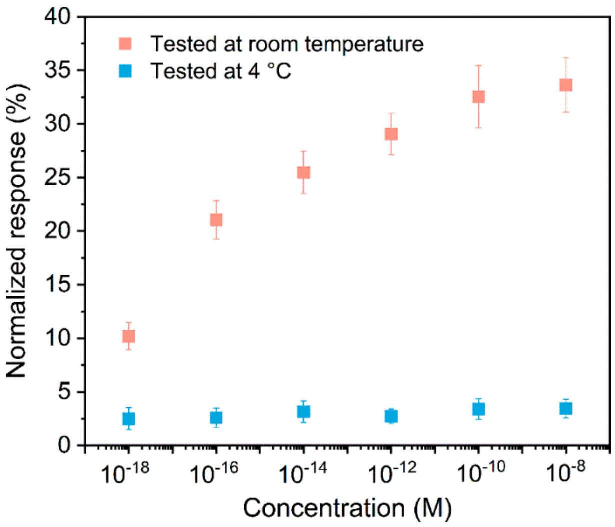

Figure S6. Concentration-dependent sensor responses measured at room temperature and 4 °C (n = 5, mean ± SE).

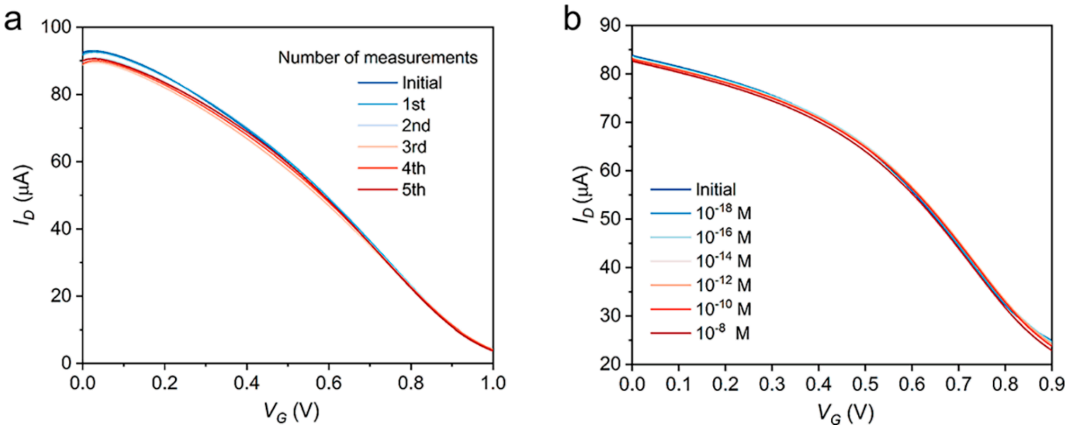

Figure S7. Device stability test. (a) Transfer curves after five consecutive additions of 0.1× PBS. (b) Transfer curves of the non-functionalized gate sensor after addition of S protein.

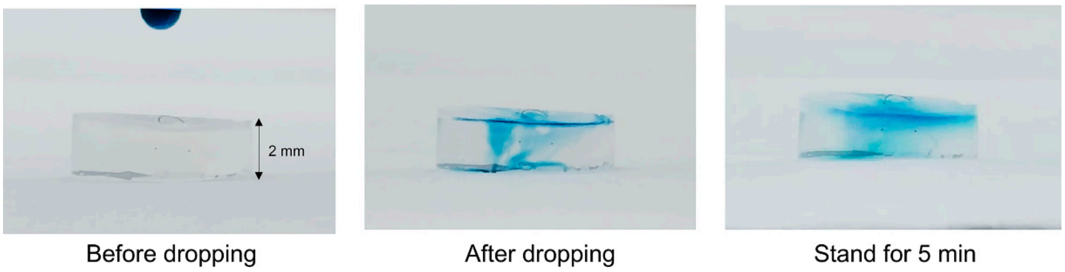

Figure S8. Penetration experiment of solution in the 0.5 wt% agarose hydrogel. Methylene blue solution (0.1%) was dropped onto agarose hydrogel with a thickness of 2 mm.

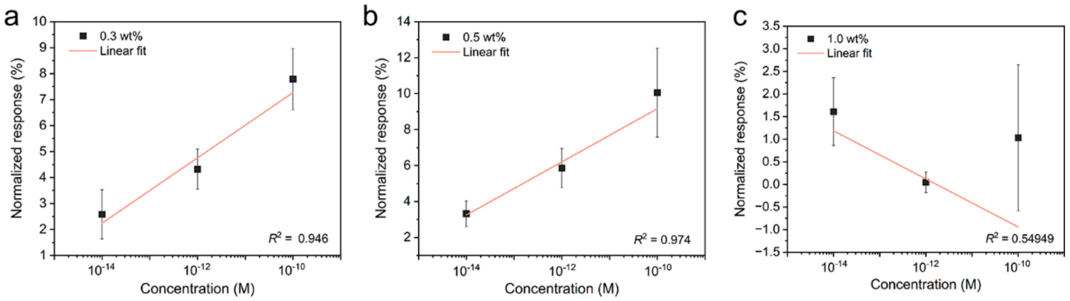

Figure S9. Linear fitting of S protein responses measured with hydrogels of different concentrations. (a) 0.3 wt%. (b) 0.5 wt%. (c) 1 wt%.

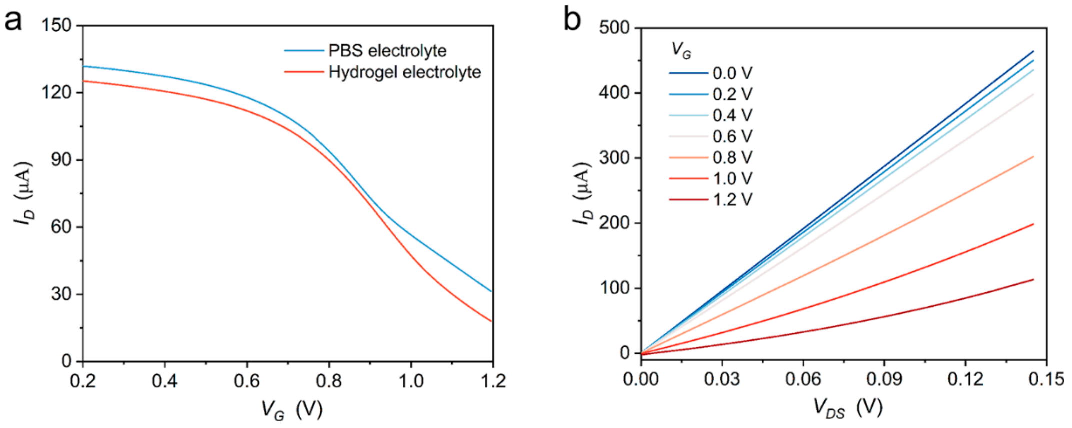

Figure S10. (a) Comparison of transfer curves measured with hydrogel and 0.1× PBS electrolytes. (b) Output characteristics measured in 0.1× PBS.

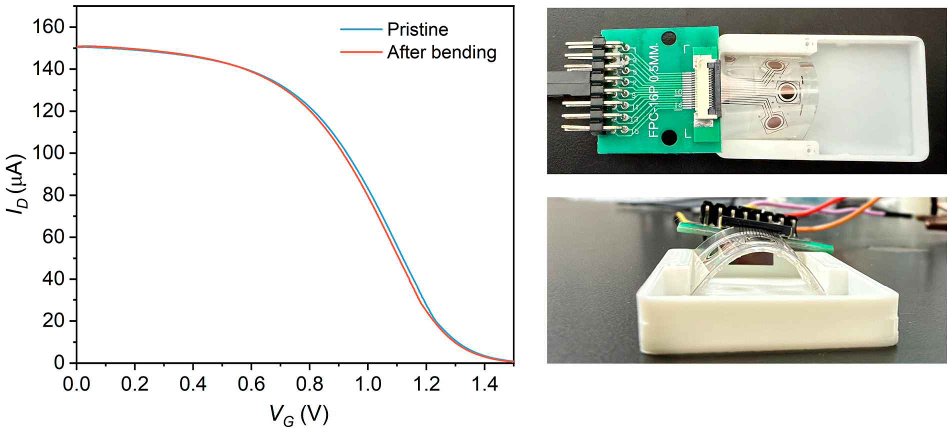

Figure S11. Comparison of the OECT transfer characteristics measured in the flat and bent states. Photographs show the device in the corresponding measurement configurations.

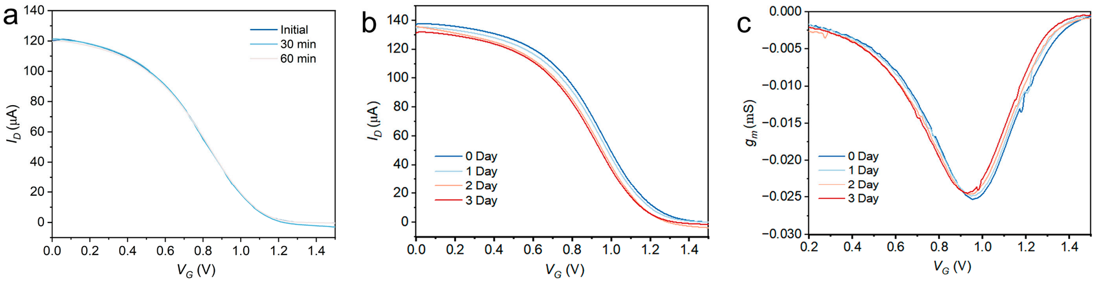

Figure S12. Temporal stability of aptamer-functionalized, hydrogel-coated OECTs. (a) Transfer characteristics measured initially and after 30 and 60 min of exposure to ambient air. (b) Transfer characteristics measured on days 0–3 during sealed storage at 4 °C. (c) Transconductance curves calculated from the transfer characteristics in (b).

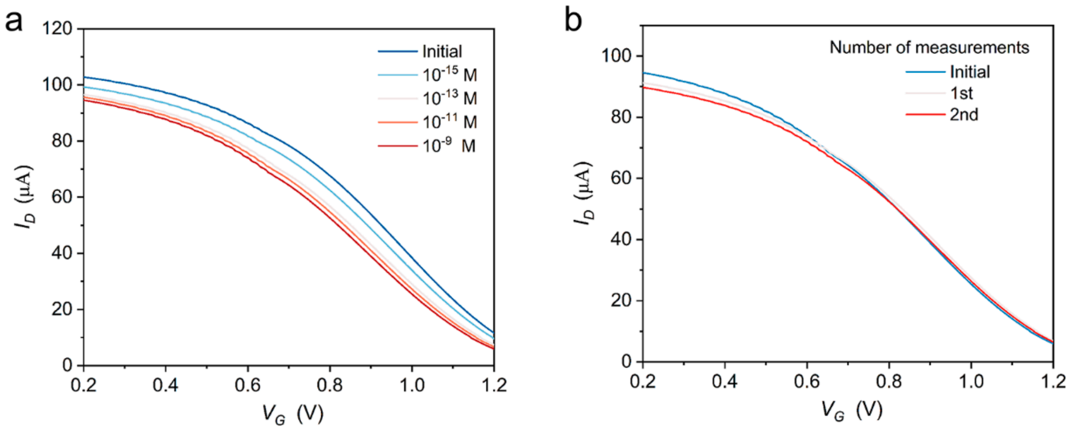

Figure S13. (a) Transfer curves measured under a 0.5 V electric field. (b) Blank control.

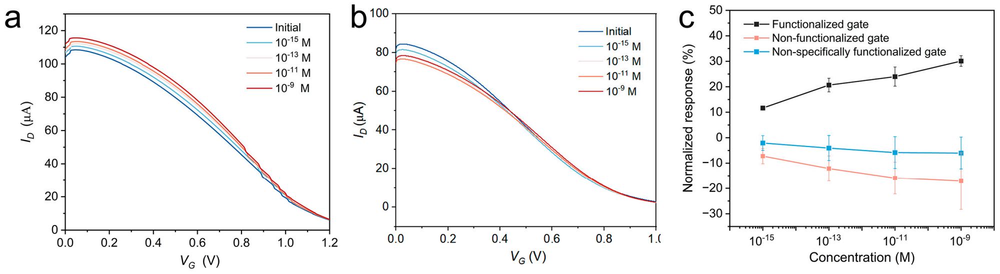

Figure S14. Specificity controls for SARS-CoV-2 spike protein detection. Representative OECT transfer characteristics obtained after exposure to increasing concentrations of spike protein using (a) a non-functionalized gate and (b) a gate modified with a non-specific thiolated DNA control (5′-SH-C_6-TTTTTTTTTT GGTGCTGGAGAG-3′). (c) Comparison of the normalized responses of the aptamer-functionalized, non-functionalized, and non-specific-DNA-functionalized gates. All devices were evaluated using the same 0.5 V field-assistance and subsequent OECT-readout protocol (n = 3; mean ± SE).

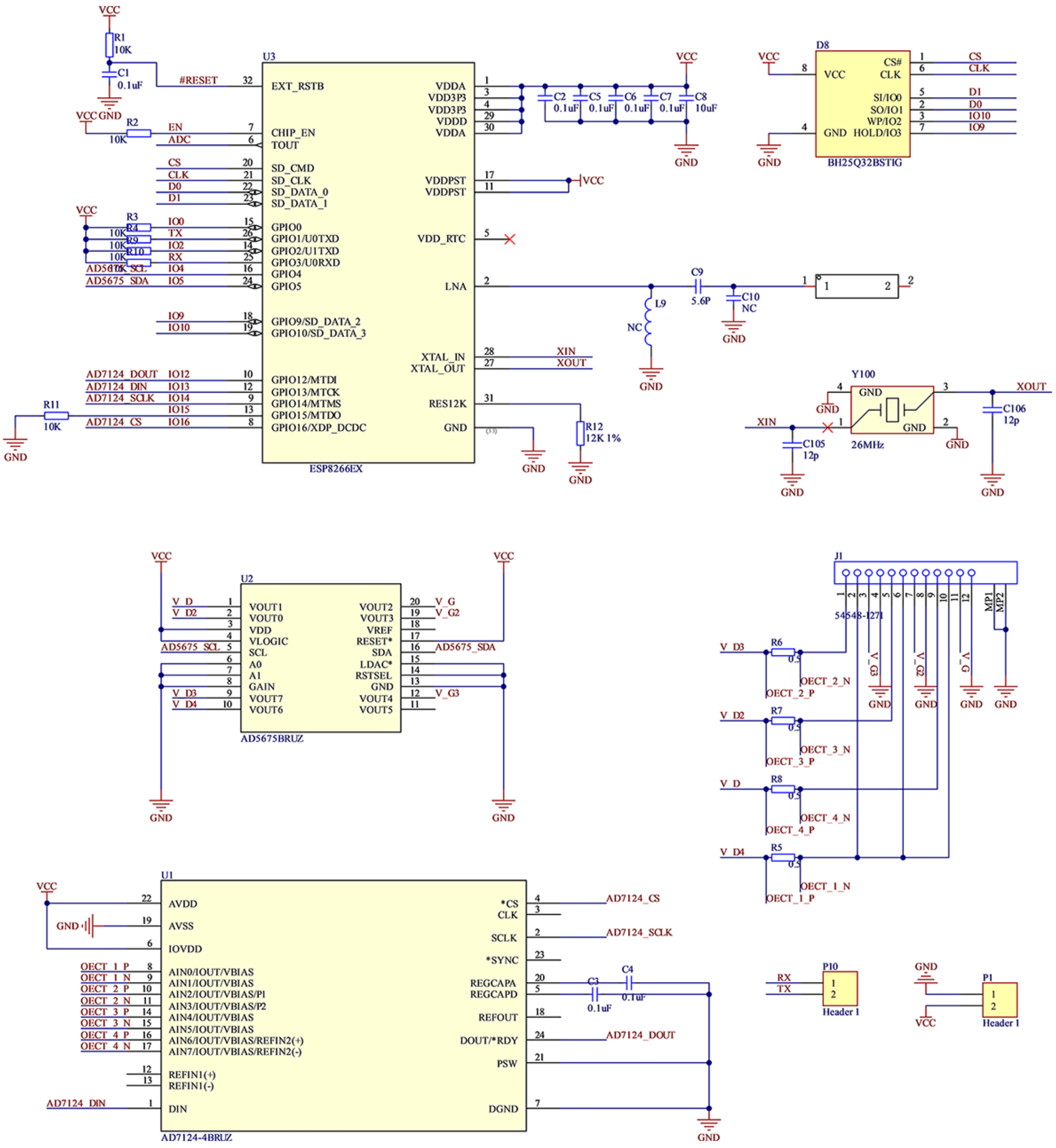

Figure S15. Circuit diagram of the portable device.

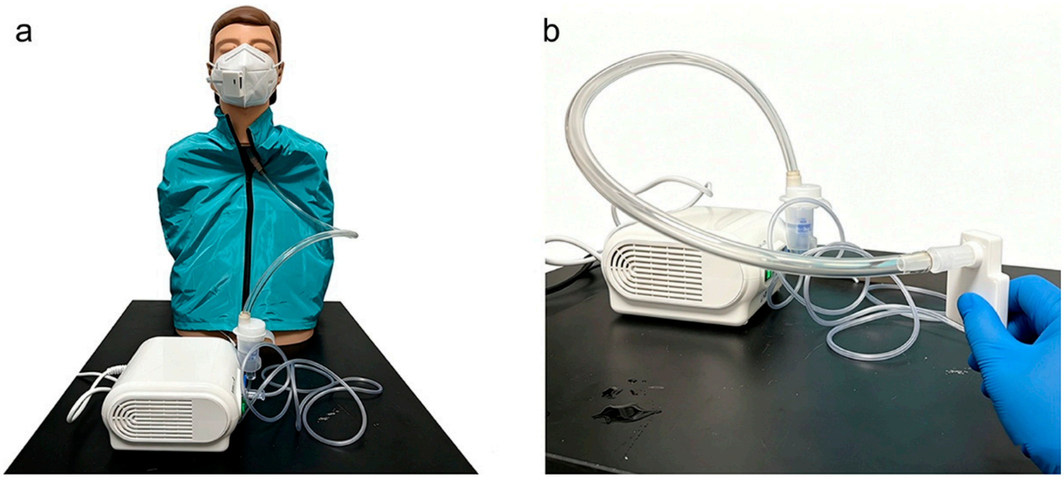

Figure S16. Nebulizer-based simulation test. (a) Face-mask-based test. (b) Handheld test.

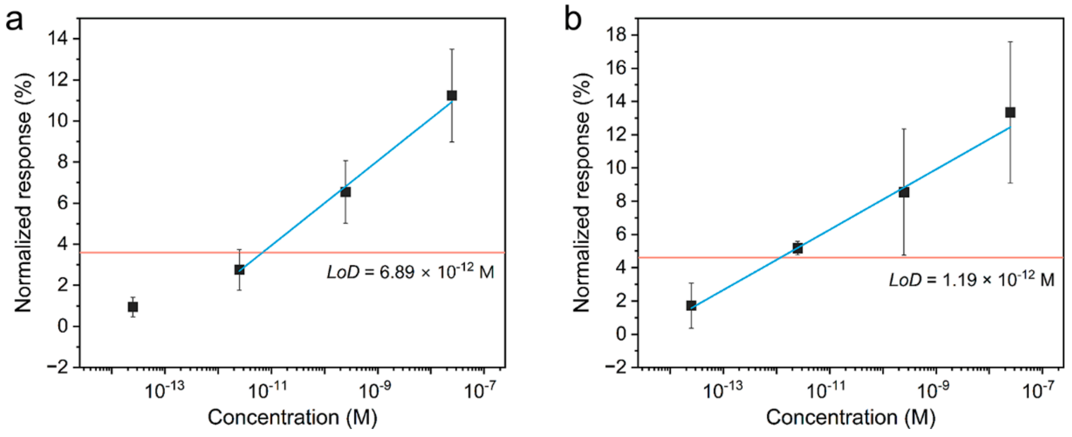

Figure S17. Fitted calibration curve for concentration-dependent responses under nebulizer-based testing. (a) Face-mask-based test, $R^2 = 0.996$. (b) Handheld test, $R^2 = 0.994$. n = 3; mean ± SE.

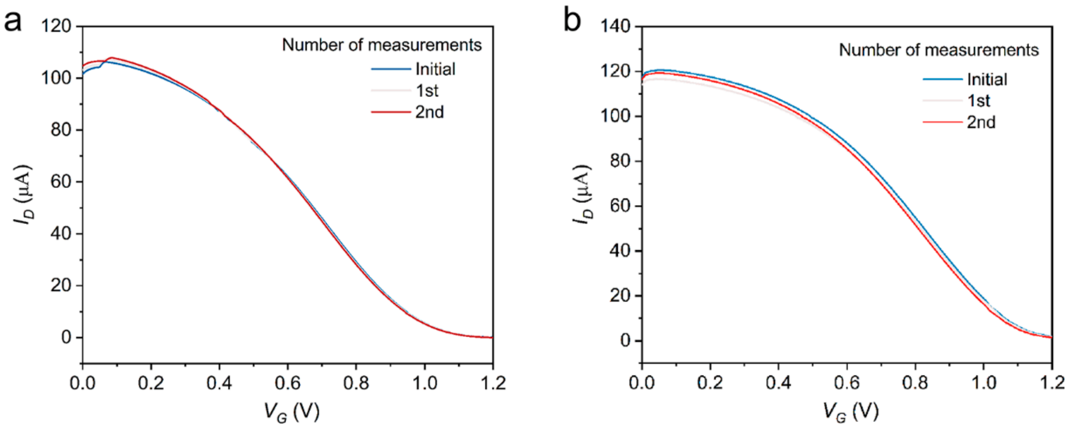

Figure S18. Blank control: nebulization test using 0.1× PBS solution. (a) Face-mask-based test. (b) Handheld test.

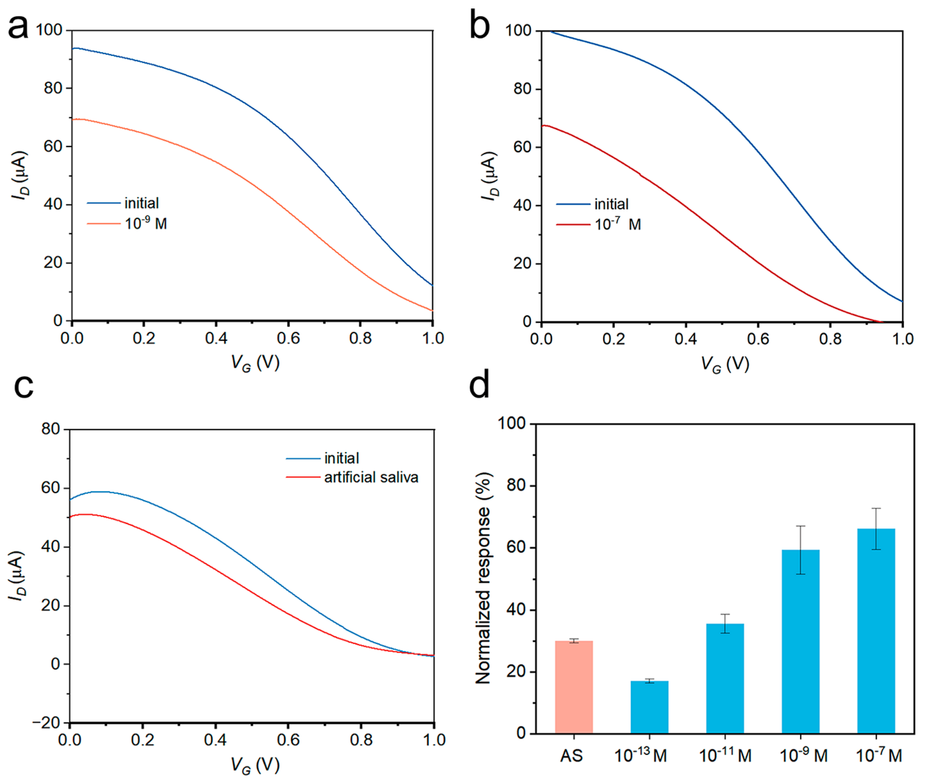

Figure S19. Detection of SARS-CoV-2 spike antigen in artificial saliva. Representative OECT transfer curves before and after exposure to nebulized artificial saliva containing (a) 10^{-9} M or (b) 10^{-7} M spike antigen, and (c) antigen-free artificial saliva. (d) Normalized responses to antigen-free artificial saliva (AS) and artificial saliva containing spike antigen at the indicated concentrations.
